# Comparison of Centor and McIsaac scores in primary care: a meta-analysis over multiple thresholds

**DOI:** 10.3399/bjgp20X708833

**Published:** 2020-03-10

**Authors:** Brian H Willis, Dyuti Coomar, Mohammed Baragilly

**Affiliations:** Institute of Applied Health Research, University of Birmingham, Birmingham.; Institute of Applied Health Research, University of Birmingham, Birmingham.; Institute of Applied Health Research, University of Birmingham, Birmingham.; Department of Applied Statistics, Helwan University, Cairo, Egypt.

**Keywords:** Centor score, diagnosis, McIsaac score, meta-analysis, pharyngitis, primary health care

## Abstract

**Background:**

Centor and McIsaac scores are both used to diagnose group A beta-haemolytic streptococcus (GABHS) infection, but have not been compared through meta-analysis.

**Aim:**

To compare the performance of Centor and McIsaac scores at diagnosing patients with GABHS presenting to primary care with pharyngitis.

**Design and setting:**

A meta-analysis of diagnostic test accuracy studies conducted in primary care was performed using a novel model that incorporates data at multiple thresholds.

**Method:**

MEDLINE, EMBASE, and PsycINFO were searched for studies published between January 1980 and February 2019. Included studies were: cross-sectional; recruited patients with sore throats from primary care; used the Centor or McIsaac score; had GABHS infection as the target diagnosis; used throat swab culture as the reference standard; and reported 2 × 2 tables across multiple thresholds. Selection and data extraction were conducted by two independent reviewers. QUADAS-2 was used to assess study quality. Summary receiver operating characteristic (SROC) curves were synthesised. Calibration curves were used to assess the transferability of results into practice.

**Results:**

Ten studies using the Centor score and eight using the McIsaac score were included. The prevalence of GABHS ranged between 4% and 44%. The areas under the SROC curves for McIsaac and Centor scores were 0.7052 and 0.6888, respectively. The *P*-value for the difference (0.0164) was 0.419, suggesting the SROC curves for the tests are equivalent. Both scores demonstrated poor calibration.

**Conclusion:**

Both Centor and McIsaac scores provide only fair discrimination of those with and without GABHS, and appear broadly equivalent in performance. The poor calibration for a positive test result suggests other point-of-care tests are required to rule in GABHS; however, with both Centor and McIsaac scores, a score of ≤0 may be sufficient to rule out infection.

## INTRODUCTION

Pharyngitis is one of the most common reasons for consulting a GP. Over the winter period, around 6% of GP consultations in the UK tend to be for patients presenting with a sore throat, which represents more than 3.5 million consultations.^1^ Although, in many cases, pharyngitis has a viral aetiology, 20%–35% of cases may be caused by bacteria — specifically, group A beta-haemolytic streptococcus (GABHS).^2^^,^^3^ Worldwide, infection with group A streptococci (GAS) places a significant burden on global health, and around 500 million people will die from GAS-related diseases each year.^4^

In order to stratify patients most at risk of GABHS, the Centor score was developed. Each of four clinical features — absence of cough, purulent pharyngeal exudate, anterior cervical lymphadenopathy, and temperature of >38°C — is scored with 1 or 0, depending on whether it is present;^5^ scores range from 0 (when none of the features are present) to 4 (when all are present). In the original study, conducted in an emergency department in the US, a score of 3 was associated with a 30.1%–34.1% probability of GABHS.^5^ McIsaac independently derived a prediction system based on a cohort of patients from primary care.^6^ In essence, it modifies the Centor system to include an extra variable — age. For those aged between 3 years and 14 years, 1 is added to the score, whereas, for those aged ≥45 years, 1 is subtracted from the score; hence, a patient presenting with a sore throat may have a McIsaac score of anything between −1 and 5.^6^

Many health systems have recommended the use of Centor or McIsaac scores in their guidelines to help manage patients with acute pharyngitis.^7^^–^^10^ In the UK, the Centor score is one of two prediction rules recommended by the National Institute for Health and Care Excellence (NICE).^10^ Although the extent to which these rules are used in UK general practice is unclear, a recent survey of 266 GPs in Denmark reported that approximately half used the Centor score and 15% used the McIsaac score — this was in spite of the fact that the McIsaac score is the recommended rule in Denmark for diagnosing GABHS.^9^

The question of which rule is likely to yield the most accurate diagnosis of GABHS for patients presenting to general practice is difficult to answer based on existing research. Only one primary study to date — reported in two articles by Fine *et al* ^11^^,^^12^ — provides the data to allow a direct comparison. Furthermore, comparisons at individual thresholds are meaningless, as those thresholds are not equivalent to each other — for example, a Centor score of 3 is not equivalent to a McIsaac score of 3, as the latter is calculated with an extra variable (that of age). To compare the tests, an overall assessment across all thresholds is required, such as may be provided by a receiver operating characteristic (ROC) curve.

Although meta-analysis allows the aggregation of multiple studies, either to produce a summary (sensitivity, false positive rate) point or a summary ROC curve,^13^^–^^15^ both of these methods are constrained by the inclusion of only one (sensitivity, false positive rate) data point per study, where the false positive rate = 1 − specificity. When a study reports data at multiple thresholds, an arbitrary choice has to be made on which threshold to use when extracting the data for meta-analysis. Recent developments in meta-analysis methods allow this constraint to be relaxed so, if individual studies provide data at multiple thresholds, all of the data may be included for analysis;^16^ as such, the unit of interest for each study becomes its ROC curve and not just an individual (sensitivity, false positive rate) pair. This provides the basis for generating a summary ROC (SROC) curve for the Centor and McIsaac scores based on all the data reported in the primary studies.

**Table:** How this fits in

In many healthcare systems, the Centor score and McIsaac score are used by GPs and primary care professionals to diagnose group A beta-haemolytic streptococcus (GABHS); however, there is no previous meta-analysis that has compared their performances in primary care. This comparative meta-analysis demonstrates that the Centor score and McIsaac score have broadly similar performance characteristics in diagnosing GABHS infection in primary care. A score of ≤0 when using either system may have a role in ruling out GABHS infection in primary care; however, neither score is sufficiently accurate to rule in GABHS infection and, if applied as recommended, could lead to more than one in two patients being prescribed antibiotics inappropriately. Other point-of-care diagnostics that augment these scores are needed if rates of inappropriate antibiotic prescribing are to be reduced.

This study aimed to compare the performance of Centor and McIsaac scores in diagnosing patients with GABHS presenting to primary care with a sore throat.

## METHOD

### Data sources and searches

MEDLINE, EMBASE, and PsycINFO were searched for relevant studies; the search terms used are given in Supplementary Box S1. The data were supplemented by a manual review of the references from two published meta-analyses — one by Aalbers *et al*,^17^ the other by Willis and Hyde.^18^ The grey literature was not specifically searched because of a lack of evidence supporting its use in test accuracy reviews;^19^^,^^20^ however, for completeness, a Google Scholar search was also performed using the terms ‘McIsaac score’ and ‘Centor score’. The searches were limited to studies published between January 1980 and February 2019. No restrictions were placed on the language of publication. Duplicate references were discarded to get a cohesive set of studies ready to be reviewed for inclusion.

### Study eligibility criteria

Studies were included if:

- the study was a cross-sectional primary study;
- the study population consisted of unselected patients presenting with a sore throat to primary care;
- the study evaluated at least one of Centor or McIsaac scores;
- the target diagnosis was GABHS;
- the reference standard was culture from a throat swab; and
- sufficient data were reported to complete the 2 × 2 table for as many thresholds as possible.

Two researchers independently screened the title and abstracts of all citations identified. Full texts were obtained for those articles not excluded at the screening stage, and the same two investigators independently assessed the studies for eligibility based on the above criteria. Disagreements were resolved through discussion and achieving consensus.

### Data collection and quality assessment

Data were extracted on the following study characteristics:

- aim;
- test evaluated;
- start and end date;
- method of subject recruitment;
- study location;
- description of study population;
- sample size;
- reference standard;
- conclusion of study authors;
- 2 × 2 contingency table data (true positives, false positives, true negatives, and false negatives) for each reported threshold on a per-patient basis; and
- any conflicts of interest.

The quality of each included study was assessed using the Quality Assessment of Diagnostic Accuracy Studies (QUADAS-2) tool,^21^ which assesses the risk of bias across a number of domains. The category ‘unclear’ was used when there was insufficient information reported in the study to come to a clear decision even after discussion.

The same two researchers who screened the initial abstracts independently extracted data, and performed the appraisal and quality assessment of each study. Disagreements were resolved through discussion and achieving consensus.

### Synthesis and meta-analysis methods

The Different random Intercept Different random Slope (DIDS) model from the R package diagmeta (https://CRAN.R-project.org/package=diagmeta) was used to fit the data from the primary studies. This fits two linear mixed models — one for the false negative rate and one for the specificity — using the study as the grouping factor and allowing data from multiple thresholds for each study. Each linear mixed model has a random intercept and random gradient term, and the four random effects are assumed to have a four-dimensional multivariate normal distribution;^16^ these are used to generate an SROC curve.

An SROC curve and C-statistic (area under the curve [AUC]) was generated for Centor and McIsaac scores. Positive and negative likelihood ratios were derived for each of the thresholds with bootstrap confidence intervals (CIs). Assuming a null hypothesis that there is no difference between the C-statistics, the null distribution was derived empirically using a bootstrap sample of 1000. The level of significance was set to 0.05. For each test, the summary (sensitivity, false positive rate) pair corresponding to each threshold was also derived. Calibration plots of expected probabilities versus observed probabilities were derived for positive and negative test results after fitting an additive model to the logits of these probabilities using cubic splines.^22^ Each plot was corrected for optimism using a bootstrap sample of 1000 as recommended by Harrell.^23^

## RESULTS

### Study selection

**Figure 1.**
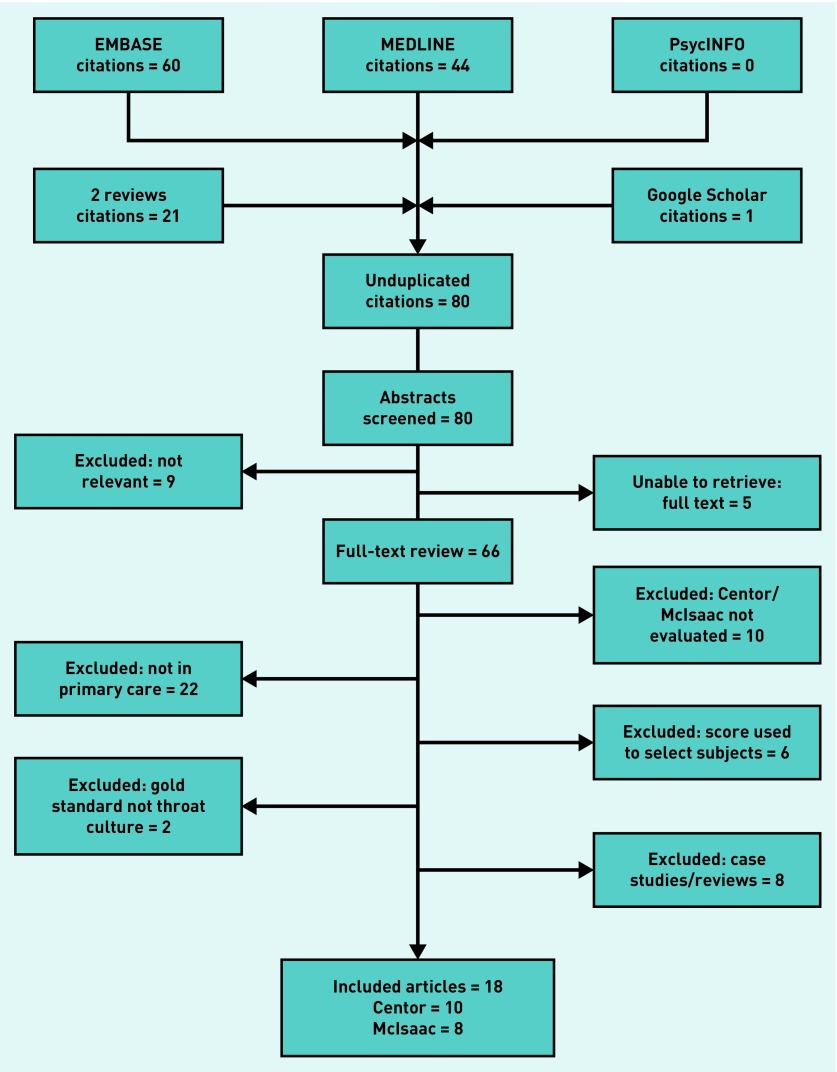
***Flow chart of studies selected.***

The searches identified 80 citations. The full selection process (outlined in Figure 1) resulted in 18 studies^2^^,^^6^^,^^11^^,^^12^^,^^24^^–^^37^ being included in the review; 10 of these used the Centor score^2^^,^^11^^,^^12^^,^^24^^–^^31^ and eight used the McIsaac score.^6^^,^^11^^,^^12^^,^^32^^–^^37^ Only one study — reported by Fine *et al* ^11^^,^^12^ — provided sufficient data to allow a direct comparison between the two tests.

A flowchart of the primary studies’ selection decisions is given in Figure 1.

### Study characteristics

Full study characteristics are detailed in Table 1. Of those studies using the Centor score, eight were conducted in Europe^2^^,^^24^^–^^27^^,^^29^^–^^31^ and two in the US.^11^^,^^12^^,^^28^ Of those studies using the McIsaac score, three were conducted in Europe,^32^^,^^33^^,^^36^ four in North America,^6^^,^^11^^,^^12^^,^^35^^,^^37^ and one in Australia.^34^ Three studies were translated from Spanish.^26^^,^^31^^,^^36^ The only study to provide data on both the Centor and McIsaac scores (Fine *et al* ^11^^,^^12^) had a sample size that was more than 100 times larger than the next-largest study.

**Table 1.** Study characteristics

**Study**	**Location**	**Score**	**Year**	**Setting**	**Age, years**	**Sample**	**Prevalence, %**	**Thresholds**	**Reference standard**
Alper^24^	Bursa, Turkey	Centor	May 2007 to Apr 2008	Emek Family Practice Centre	7–86	282	11.4	0,1,2,3,4	Throat culture
Fine^11^^,^^12^	26 states in US	Centor/McIsaac	1 Sep 2006 to 1 Dec 2008	Around 581 minute clinics in CVS chain across 26 states	3–≥55	206 870	27.1	0,1,2,3,4 −1,0,1,2,3,4,5	DNA probe and throat culture for RADT negatives, RADT for test positives
Little^25^	UK	Centor	Jan 2007 to Oct 2008	General practices	≥5	1086	33.7	0,1,2,3,4	Throat culture
Marin Cañada^26^	Madrid, Spain (Spanish)	Centor	14 Feb 2005 to 12 May 2005	San Fernando 2 Health Centre	14–81	140	24.2	0,1,2,3,4	Throat culture
Lindbæk^27^	Stokke & Kongsberg, Norway	Centor	Apr 2000 to Jun 2002	1 general practice in Stokke and 1 in Kongsberg	Children and adults	300	42.0	0,1,2,3,4	Throat culture
Atlas^28^	Massachusetts, US	Centor	1 Jul 2002 to 30 Jun 2003	2 primary care centres in Massachusetts General Hospital	Adults	148	25.7	0,2,3,4	Throat culture
Chazan^29^	Nazareth, Israel	Centor	Dec 1999 to Mar 2000	Primary care clinics of the Clalit Health Services	16–80	204	24.5	0,2,4	Throat culture
Seppälä^30^	Turku, Finland	Centor	Jan 1986 to Mar 1986	Private health centre Pulssi	15–62	106	4.7	0,3,4	Throat culture
Regueras De Lorenzo^31^	Asturias, Spain (Spanish)	Centor	Jan 2008 to May 2010	5 primary care centres	2–14	192	38.5	0,3	Throat culture
Dagnelie^2^	Utrecht, Netherlands	Centor	1990 to 1992	53 GPs in general practice	4–60	558	32.8	0,3	Throat culture
Stefaniuk^32^	Warsaw, Poland	McIsaac	Mar 2014 to May 2014	Orlik General Practice	1–≥40	96	44.8	1,2,3,4,5	Throat culture
Mistik^33^	Kayseri, Turkey	McIsaac	Jun 2013 to Jun 2014	Bunyamin Somyurek Family Medicine Centre	3–85	624	12.7	−1,0,1,2,3,4,5	Throat culture
Dunne^34^	Melbourne, Australia	McIsaac	Winter/spring of 2011 and 2012	3 general practices & ED in tertiary hospital	3–72	127	18.9	−1,1,2,3,4	Throat culture and PCR
Tanz^35^	Chicago and Cincinnati, US	McIsaac	15 Nov 2004 to 15 May 2005	6 community-based paediatric offices	3–18	1848	29.9	0,1,2,3,4,5	Throat swab culture, 6 had RADT
Flores Mateo^36^	Barcelona, Spain (Spanish)	McIsaac	Mar 2008 to May 2009	2 primary care centres in Castelldefels	1–14	211	34.1	0,3,4,5	Throat culture
McIsaac^37^	Ontario, Canada	McIsaac	Oct 1998 and Mar 1999	97 family physicians from 49 communities	Children and adults	580	17.2	−1,1,2,3,4	Throat culture
McIsaac^6^	Toronto, Canada	McIsaac	Dec 1995 to Feb 1997	University-affiliated family medicine centre	3–76	503	12.9	−1,1,2,3,4	Throat culture

ED = emergency department. PCR = polymerase chain reaction. RADT = rapid antigen detection test.

**Figure 2.**
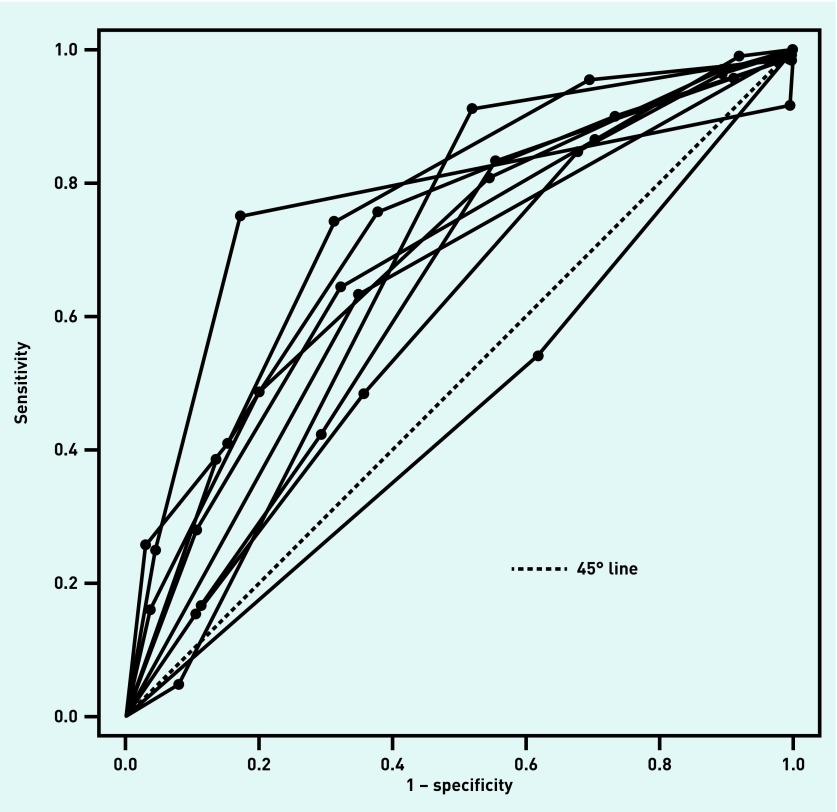
***Centor score: ROC curves for each of the included studies. Each line corresponds to a single study and each dot corresponds to the (sensitivity, 1 − specificity) pair at a particular threshold for that study. ROC = receiver operating characteristic.***

**Figure 3.**
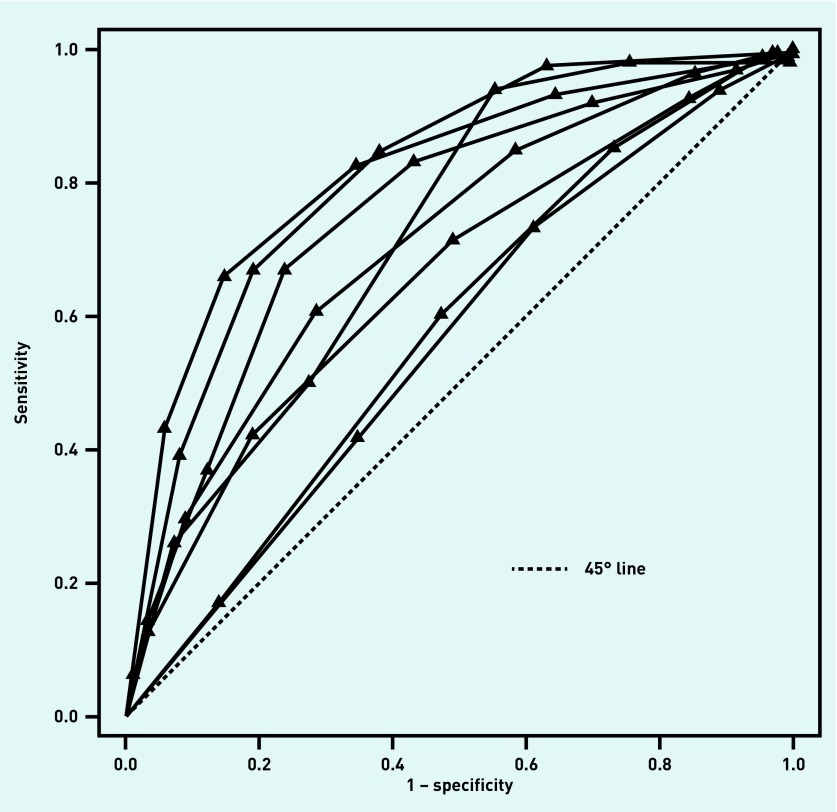
***McIsaac score: ROC curves for each of the included studies. Each line corresponds to a single study and each triangle corresponds to the (sensitivity, 1 − specificity) pair at a particular threshold for that study. ROC = receiver operating characteristic.***

The median prevalence of GABHS for the studies using the Centor score was 26.4% (range: 4.7%–42.0%); for studies using the McIsaac score, it was 23.0% (range: 12.7%–44.8%). Exactly half of the studies using the Centor score provided data on all thresholds, and all studies provided data for two or more thresholds. A quarter of studies using the McIsaac score provided data on all thresholds, and all studies provided data for ≥4 thresholds. The ROC curves for each of the studies using the Centor score are shown in Figure 2; those for each of the studies using the McIsaac score are given in Figure 3.

For two of the included studies using the McIsaac score, McIsaac was listed as the lead author.^6^^,^^37^

### Risk of bias and applicability

There is no validated statistic for measuring between-study heterogeneity across ROC curves; however, Figure 2 and Figure 3 show that, for both tests, the ROC curves are widely distributed; this suggests there is heterogeneity between studies for both tests.

For many of the studies,^2^^,^^6^^,^^25^^–^^28^^,^^30^^–^^35^^,^^37^ the reporting was inadequate, which introduced uncertainty when assessing the risk of bias — for example, the method of patient selection was not always described and it was not always clear whether any subjects had been excluded. Often, it was not reported whether the reference standard was carried out blind to the test results, although it is unclear whether knowledge of the test results would have greatly influenced the results of a cultured throat swab. In general, the study populations were considered representative of those seen in the different forms of primary care.

In two studies^31^^,^^37^ there were discrepancies between the number of subjects recruited and the number used in analyses, thereby increasing risk of biased estimates for the statistics of interest. In addition, although in one study that used the Centor score the target condition was largely GABHS, it also included group C and group G streptococcal infection as the target condition.^25^ This could potentially affect the applicability of the findings of this study. QUADAS-2 results are given in Supplementary Figure S1 (McIsaac score) and Supplementary Figure S2 (Centor score).

### Synthesis of results

**Figure 4.**
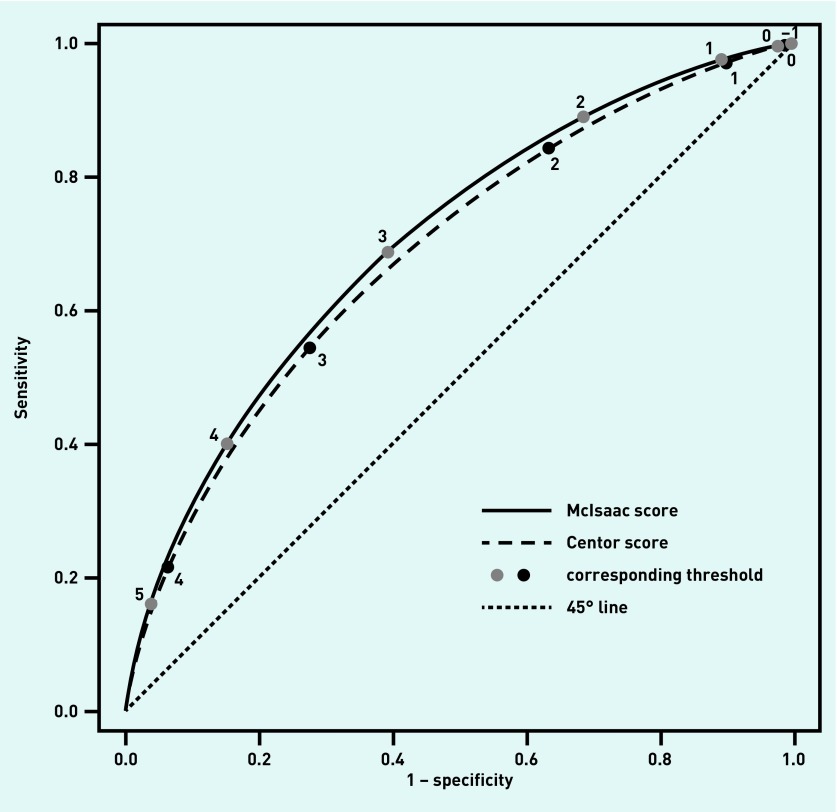
***SROC curves for the McIsaac and Centor scores. SROC = summary receiver operating characteristic.***

The sensitivities, specificities, and positive and negative likelihood ratios for each threshold are given for both scores in Table 2. Figure 4 shows the SROC curves for the Centor and McIsaac scores, with points on each curve corresponding to particular thresholds; it is clear that the curves are very close to each other and this is confirmed by the C-statistic. For the Centor score, the C-statistic was 0.6888 (95% CI = 0.653 to 0.724) and for McIsaac’s score it was 0.7052 (95% CI = 0.624 to 0.778); the 95% CIs are for the sensitivity given the specificity. From the empirical distribution of the difference between C-statistics, a difference of 0.0164 has a corresponding *P*-value of 0.419; this suggests there is no statistically significant difference between the C-statistics for the two curves.

**Table 2.** Sensitivity and specificity of the Centor and McIsaac scores at different thresholds

**Threshold**	**Sensitivity, % (95% CI)**	**Specificity, % (95% CI)**	**LR+ (95% CI)**	**LR− (95% CI)**
**Centor score**				
1	97.2 (96.4 to 97.8)	10.1 (6.3 to 15.2)	1.08 (1.05 to 1.14)	0.28 (0.23 to 0.45)
2	84.4 (81.4 to 87.0)	36.7 (28.8 to 45.1)	1.33 (1.19 to 1.50)	0.43 (0.39 to 0.55)
3	54.4 (48.7 to 60.0)	72.4 (64.4 to 79.4)	1.97 (1.46 to 2.40)	0.63 (0.58 to 0.74)
4	21.5 (16.6 to 27.2)	93.7 (89.6 to 96.4)	3.41 (1.83 to 4.97)	0.84 (0.78 to 0.90)

**McIsaac score**				
0	99.7 (99.0 to 99.9)	2.3 (0.3 to 10.7)	1.02 (1.00 to 1.10)	0.15 (0.09 to 0.35)
1	97.5 (94.7 to 99.0)	10.8 (2.8 to 28.4)	1.09 (1.02 to 1.30)	0.23 (0.17 to 0.39)
2	88.0 (82.0 to 93.8)	31.5 (14.2 to 54.3)	1.30 (1.09 to 1.76)	0.35 (0.28 to 0.47)
3	68.7 (57.4 to 78.5)	60.8 (39.9 to 78.8)	1.75 (1.28 to 2.79)	0.51 (0.44 to 0.58)
4	40.0 (28.5 to 52.5)	84.8 (70.8 to 93.4)	2.64 (1.68 to 4.95)	0.71 (0.62 to 0.78)
5	16.1 (8.9 to 26.2)	96.3 (90.8 to 98.7)	4.32 (2.38 to 10.0)	0.87 (0.79 to 0.93)

CI = confidence interval. LR+ = positive likelihood ratio. LR− = negative likelihood ratio.

Two post-hoc sensitivity analyses were carried out. The first investigated the effect of excluding the largest study (that by Fine *et al* ^11^^,^^12^ and resulted in the C-statistics for the Centor and McIsaac scores being 0.6724 (95% CI = 0.610 to 0.731) and 0.7167 (95% CI = 0.632 to 0.788), respectively. As such, the effect is to decrease the C-statistic for the Centor score and to increase it for the McIsaac score. Again, the difference (0.0443) was not statistically significant (*P* = 0.188). In the second analysis, it was noted that two of the eight included studies that used the McIsaac score were led by the researcher who proposed it (namely, McIsaac);^6^^,^^37^ as such, only six studies evaluated the score independently. A sensitivity analysis was conducted in which the two studies led by McIsaac were excluded to evaluate the overall effects on the C-statistic. The C-statistic for the six independent studies was 0.6700 — lower than that when all studies were included in the analysis (0.7052) and that for the Centor score (0.6888).

The calibration plot for the post-test probabilities after a positive test result (positive predictive value [PPV]) for both scores, after correcting for optimism, is shown in Figure 4. The curves broadly coincide, with overfitting being particularly evident for expected PPVs above 0.5. Supplementary Figure S3 shows the calibration plot for the post-test probabilities for a negative test result after correcting for optimism. Here, the Centor score demonstrates better calibration than the McIssac score. For the derivation of both calibration plots, the prevalence of GABHS is assumed to be known.

**Figure 5.**
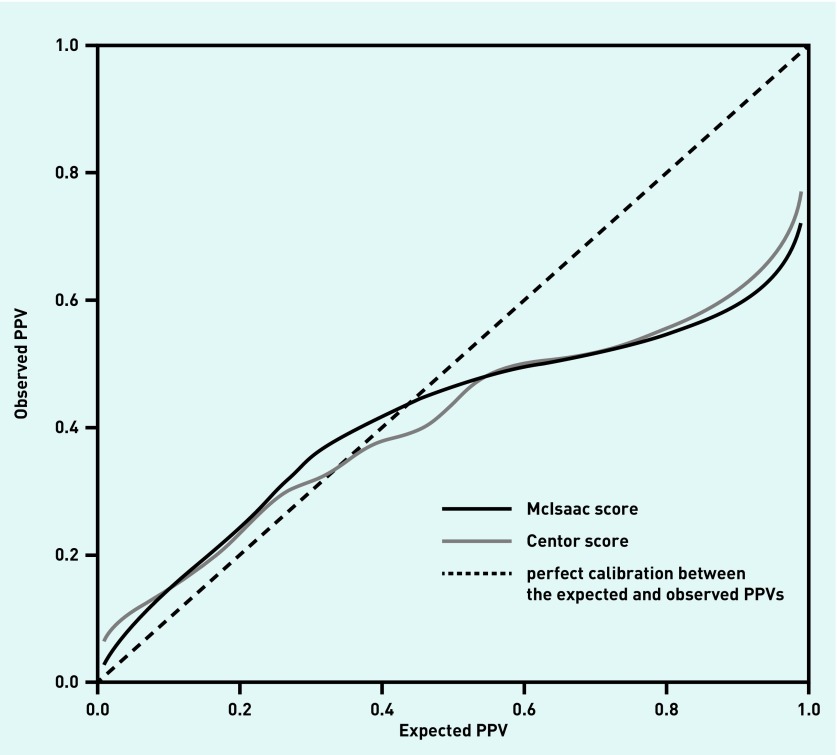
***Calibration plots (corrected for optimism) for the Centor and McIsaac scores for a positive test result when the prevalence of GABHS is assumed to be known. GABHS = group A beta-haemolytic streptococcus. PPV = positive predictive value.***

Whether either test could be used to rule in, or rule out, infection is not fully addressed by the AUC. For a GABHS infection prevalence of 25%, using Bayes’ theorem the expected PPV for a McIsaac score of 5 is 59%; however, from the calibration curve this expected PPV is likely to translate into an observed PPV of around 49% (Figure 5). Thus, if a score of 5 is used as the threshold for prescribing antibiotics, a PPV of 49% translates into more than one in two patients receiving antibiotics unnecessarily. Although the expected PPV would increase with GABHS prevalence, the calibration curves show this would not substantially affect the observed PPV; as such, neither test is effective at ruling in GABHS.

These results lead to the question of whether these criteria can be used to rule out infection. For a McIsaac score threshold of 1, a negative test corresponds to a score of −1, or 0. Similarly, at a threshold of 0, a negative test is a score of −1. From Table 2, the negative likelihood ratios (LR−) for the McIsaac score at thresholds 0 and 1 are 0.15 and 0.23, respectively. Thus, at a prevalence of GABHS infection of 25%, from Bayes theorem, low McIsaac scores such as −1 and 0 give expected probabilities of infection of 4.8% and 7.1%. Equally, for the same prevalence, a Centor score of 0 gives an expected probability of infection of 8.1%. For low probabilities, the corresponding calibration curves are more erratic for the McIsaac score than the Centor score (Supplementary Figure S3), so it is not clear how well these expected probabilities translate into practice. Nonetheless, given a shared decision between GP and patient on what constitutes an acceptable risk of GABHS, a low score on either criteria may be considered as sufficient evidence not to pursue treatment or further investigation.

## DISCUSSION

### Summary

This is the first meta-analysis to compare the performances of the Centor and McIsaac scores in a primary care setting over multiple cut points. Although there were 10 studies that evaluated the Centor score and eight that evaluated the McIsaac score, only one primary study provided data that allowed a direct comparison of the two tests.

The meta-analysis demonstrated that the SROC curves were broadly aligned, with the curve for the McIsaac score lying slightly above that for the Centor score (see Figure 4); however, the difference was marginal and no statistically significant difference between the AUCs was found. Moreover, when those studies authored by McIsaac were excluded, a sensitivity analysis revealed that the AUC for the McIsaac score may be overstated. Nonetheless, this did not alter the conclusion that the two prediction scores have similar performance characteristics and that adding an age variable does not appear to improve the accuracy of the Centor score for diagnosing GABHS in primary care. When compared with the Centor system, the McIsaac rule changes the operating points on the SROC curve rather than improving on discrimination. In addition, with AUCs of approximately 0.7, both systems appear to be, at best, fair at differentiating those patients who have GABHS from those who do not.

The calibration of the models for both scores demonstrates over-confidence, with the expected PPVs diverging substantially from the observed PPVs for probabilities of >40%. The effect of this is that an expected PPV of 80% translates into an observed PPV of 55%. Furthermore, these plots are ‘best cases’ as they are based on the prevalence of GABHS being known for the setting. When the prevalence is unknown, the average across all studies may be used; however, in the studies that were included in this review, the prevalence of GABHS ranged between 4.7% and 44.8%, so using the average prevalence would likely lead to poorer calibration as a result.

### Strengths and limitations

All of the studies provided data at ≥2 thresholds, justifying the approach of using a model that accommodates both multiple cut points and different numbers of cut points between studies. This allowed the two criteria to be compared across the whole of the ROC space. Furthermore, by using calibration plots, it was possible to provide evidence on each criteria’s likely performance in practice and when they are most likely to be useful to clinicians.

As a reference standard, the throat swab has limitations — its performance may depend on the operator and the conditions for incubation.^38^ Alternative reference standards, such as a rising titre of the antistreptolysin O (ASO) antibody, may be used, but these also vary with age, prevalence of streptococcus, and comorbidities.^38^ ASO testing is also rarely used by investigators; none of the included studies — or those excluded due to inadequacy of a reference test — used ASO testing.

The model used in this review benefits from being able to aggregate studies that provide data at multiple thresholds; however, this needs to be weighed against the necessity for continuity corrections when there are 0 cell entries in the 2 × 2 tables. Furthermore, at present, it is not clear how the DIDS model could include study-level covariates to investigate potential sources of heterogeneity.

Some authors have recommended the use of level-specific likelihood ratios.^39^ This requires defining test positives as test results that equal the threshold score only, not the threshold score and above, as is usual practice. This alternative definition of a test positive leads to an important property of a ROC curve (monotonicity) being violated;^40^ hence, with the approach used here, it is not possible to estimate level-specific likelihood ratios.

As part of internal validation, the authors used bootstrap methods to correct the calibration plots for optimism. Other methods have been proposed that use leave-one-out cross-validation to derive a validation statistic so the internal validity of the summary estimates may be assessed;^41^ it is also possible to use other information, such as the test positive rate, to derive an estimate that is tailored to the setting of interest.^42^^,^^43^ However, a shortcoming with all of these methods, including the method used here, is that they are rarely subject to external validation; without this, it is difficult to make assertions on the transferability of the results.

### Comparison with existing literature

A recent review of guidelines for diagnosing acute pharyngitis^44^ revealed that both the Centor and McIsaac prediction scores are incorporated into guidance for Europe and North America. The Centor score is one of two prediction rules recommended for managing patients with a sore throat in the UK,^10^ while, in Denmark^9^ and Germany,^8^ the McIsaac score is recommended. This demonstrates that these scores are considered relevant to the diagnosis of acute pharyngitis in a number of countries. Therefore, it is perhaps surprising that only two reviews^17^^,^^18^ have evaluated the Centor score in primary care and no systematic review has evaluated the McIssac score in primary care. None of the reviews to date have used a model that was able to accommodate data from multiple thresholds per study in the analysis. Previous reviews^17^^,^^18^ have treated each threshold separately when aggregating studies, thereby ignoring potential correlations between thresholds at a primary-study level and at an SROC curve level. Furthermore, none of the reviews have sought to establish how well the prediction rules calibrate in practice.

As a comparison, the two previous reviews on the Centor score reported positive likelihood ratios for a threshold of 3 — 2.68 (95% CI = 1.92 to 3.75)^17^ and 2.35 (95% CI = 1.51 to 3.67)^18^ — and these were inflated compared with the ratios presented here. However, the negative likelihood ratios for a threshold of 1 were comparable: 0.27 (95% CI = 0.16 to 0.46)^17^ and 0.28 (95% CI = 0.23 to 0.45).^18^

NICE has recently recommended using either the Centor or the FeverPAIN score to assess the symptoms of patients with acute pharyngitis.^10^ Although the latter was derived from a UK population, to date this is the only study on FeverPAIN^25^ and it is yet to be replicated in other independent populations; however, it is unclear whether the FeverPAIN score would lead to a marked improvement in discrimination and calibration, particularly when it shares many of the covariates of the scores that were reviewed here.

### Implications for practice

Although the Centor score showed better calibration than the McIsaac system for a negative result, perhaps of more relevance is that, for estimated probabilities of <20%, the observed probabilities of GABHS in practice, given a negative test result, are consistently lower than the corresponding estimates. On this basis, a Centor score of 0 or a McIsaac score of ≤0 is likely to correspond to an actual risk of GABHS that is lower than the expected risk of 8.5% — as such, it is likely to be sufficient to rule out infection.

For a Centor or a McIsaac score of ≥1, it is less clear how to proceed. In general, the probability of GABHS for these scores is likely to be too high (>10%) to rule out infection and too low to rule in infection. NICE’s current recommendation is that a Centor score of ≥3 is sufficient grounds to consider prescribing antibiotics either immediately or as a delayed script with advice;^10^ however, the evidence presented here suggests that neither score can realistically identify patients with an observed risk of GABHS of >50%, irrespective of the expected risk. There is the potential that these recommendations could lead to inappropriate prescribing of antibiotics in a large percentage of cases.

In all instances, the GP should weigh up the public-health need to reduce the number of inappropriate antibiotic prescriptions and the individual patient’s need to treat a potential infection. With this in mind, an honest discussion with the patient about the likely GABHS risk and the GP’s obligation not to prescribe antibiotics inappropriately before deciding on management seems the most reasonable way to proceed.

Any substantive improvement in the diagnosis of GABHS-related pharyngitis is likely to require either a new prediction system or the use of point-of-care technologies to augment the existing clinical prediction tools.^45^
